# Lateral Subtalar Dislocation with Tarsometatarsal Dislocation: A Case Report of a Rare Injury

**DOI:** 10.1155/2017/8090721

**Published:** 2017-07-06

**Authors:** Samik Banerjee, Mostafa M. Abousayed, Douglas J. Vanderbrook, Kaushik Bagchi

**Affiliations:** Division of Orthopaedic Surgery, Albany Medical Center, 43 New Scotland Avenue, Albany, NY 12206, USA

## Abstract

Dislocation of the fourth and fifth tarsometatarsal joints in conjunction with lateral subtalar dislocation is a rare occurrence. Little is known about the mechanism of injury, the appropriate treatment for this condition, and its ultimate prognosis. In this report, we describe this atypical presentation in a middle aged, otherwise healthy male who sustained a trivial twisting injury to the ankle when he slipped and fell on ice. Open reduction and K-wire fixation were necessary to affix the lateral tarsometatarsal and talonavicular joints. At one year postoperatively, he was able to return to his preinjury occupation with mild to moderate pain with prolonged walking. His Foot and Ankle Disability Index and American Orthopaedic Foot and Ankle Society scores were 64 and 65 points, respectively. Surgical intervention resulted in a stable plantigrade foot; however, the patient had early radiographic evidence of posttraumatic arthritis in the midfoot joints at one-year follow-up.

## 1. Introduction

Subtalar dislocations are relatively uncommon injuries to the foot and ankle, accounting for 1% to 2% of all dislocations and 15% of peritalar injuries. However, they have the potential to cause substantial morbidity and functional disability [1]. Most cases of isolated subtalar dislocations are caused by moderate to high energy trauma including motor vehicle accidents and falls from height [2, 3]. Medial subtalar dislocations are encountered more frequently, with various studies reporting incidences varying between 65% and 85% [4]. Lateral dislocations are less common, found in 17 to 26% of cases, with rare case reports of both anterior and poster dislocations accounting for 1% of all subtalar dislocations [4–7].

Multiple authors have reported a high frequency of associated injuries in almost 88% of cases, such as fractures of the medial and lateral malleolus, talar neck, posterior process of talus, anterior process of calcaneus, and navicular [4, 8–13]. In addition, tendon injuries involving flexor digitorum longus, flexor hallucis longus, tibialis posterior, and extensor digitorum brevis tendons have also been reported [4, 8–13]. Frequently, lateral dislocations are associated with cuboid fractures, although involvement of the anterior calcaneal process and medial and lateral talar process had also been described.

We report an extremely rare occurrence of a closed lateral subtalar dislocation with associated dorsal dislocations of the fourth and fifth tarsometatarsal (TMT) joints and fracture of the lateral cuneiform in a 52-year-old male after a low energy fall from standing height. We discuss the mechanism of injury, its appropriate treatment, and the short-term prognosis, the latter has yet to be clearly defined in literature. We affirm the recommendations of Bibbo et al. that computed tomography (CT) scans should be obtained in all subtalar dislocations to evaluate injuries not recognized by plain radiographs alone [14]. Some of these injuries may alter treatment or portend a worse prognosis.

## 2. Case Report

**Figure 1:**
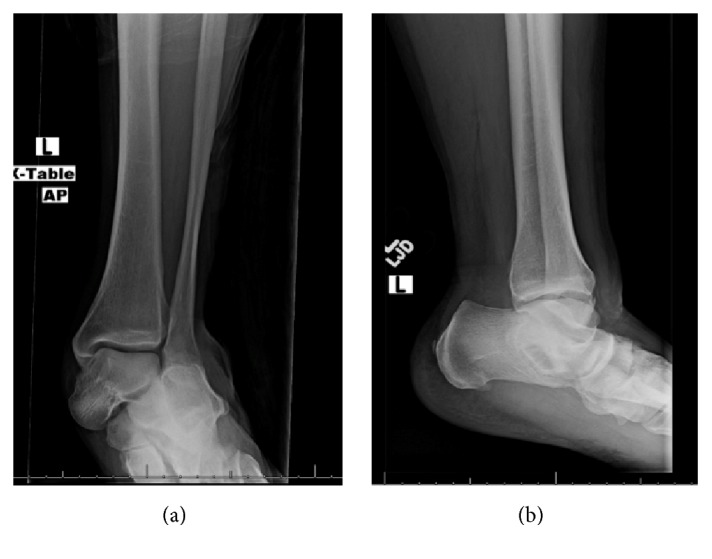
Injury radiographs demonstrating the subtalar dislocation.

A 52-year-old otherwise healthy male presented to the Emergency Department following a fall on ice from standing height sustaining a twisting injury to his left ankle. He complained of severe left ankle pain. Clinical examination revealed a moderately swollen left ankle with visible pronation deformity of the forefoot and hindfoot. Dorsalis pedis and posterior tibial pulses were intact. Sensation in the foot was preserved and all flexor and extensor tendons were intact on initial examination. Radiographs of the ankle identified a lateral subtalar dislocation with lateral displacement of the midfoot and hindfoot suggesting a subtalar dislocation (Figures 1(a) and 1(b)). Under sedation, the subtalar dislocation was successfully reduced, by placing the knee in 90 degrees of flexion and plantar-flexing the ankle, thereby relaxing the gastrocnemius-soleus complex. The hindfoot was then distracted and inverted.

**Figure 2:**
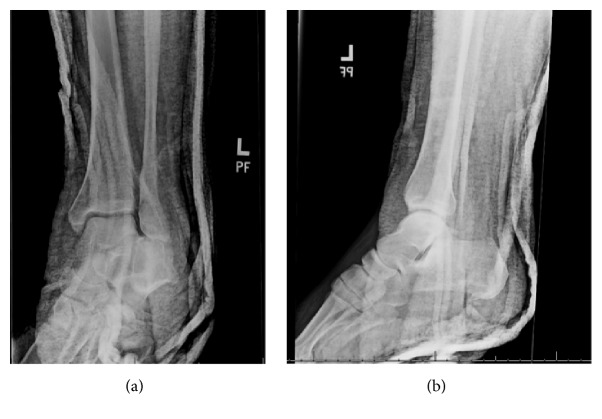
Postreduction radiographs demonstrating congruent reduction of the subtalar joint.

**Figure 3:**
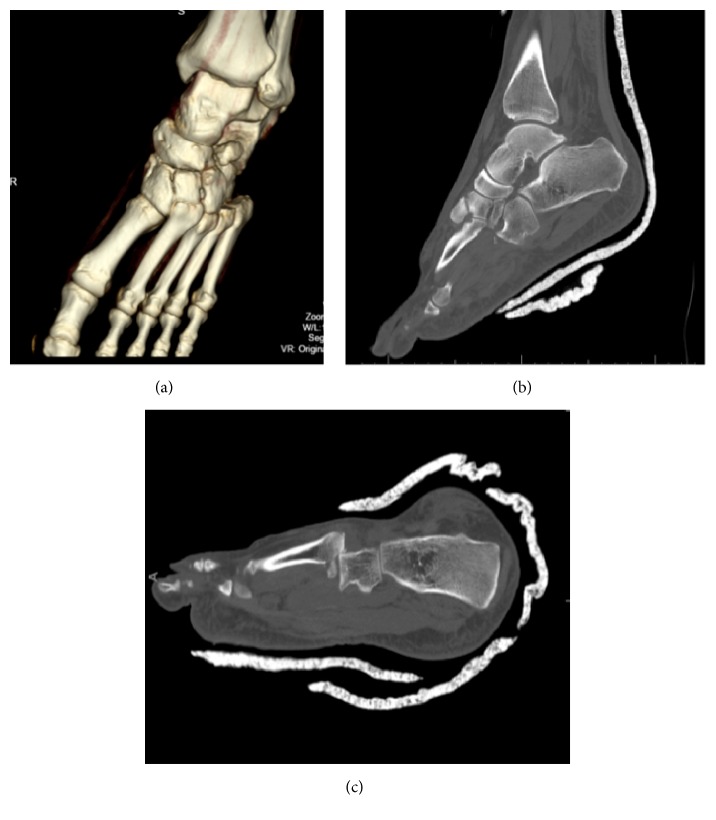
CT scan demonstrating dislocation of fourth and fifth tarsometatarsal joints.

Postreduction radiographs demonstrated a well-reduced subtalar joint (Figures 2(a) and 2(b)). However, a previously unidentified discontinuity between the base of the fourth and fifth tarsometatarsal joints was recognized. A computed tomography (CT) scan was obtained to delineate better the associated injuries and to assess for congruent reduction. CT showed a congruent reduction of the subtalar joint without evidence of any intraarticular debris. A comminuted intra-articular fracture of the base of the 4th metatarsal was found with dorsal dislocation of the fourth and fifth tarsometatarsal joints (Figures 3(a) and 3(c)). Additionally, the scans demonstrated a fracture of the plantar aspect of the lateral cuneiform.

**Figure 4:**
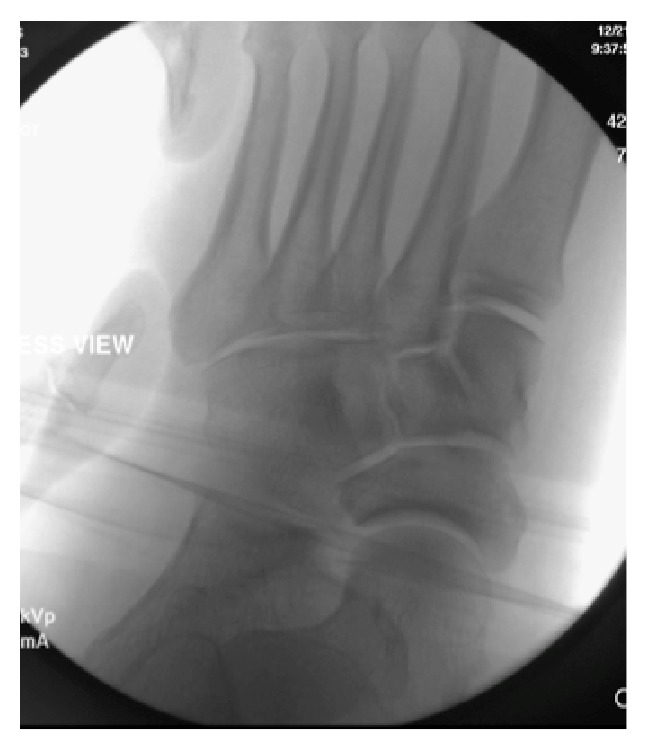
Intraoperative stress radiographs demonstrating intact first to third tarsometatarsal joints.

**Figure 5:**
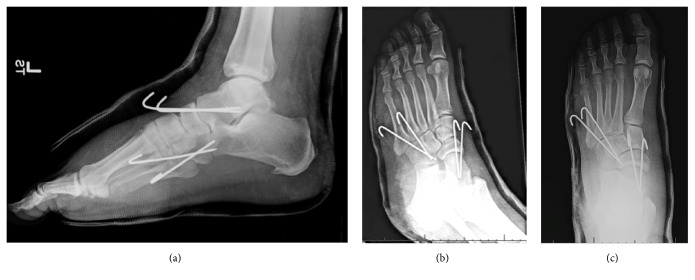
Postoperative radiographs demonstrating stabilization of the fourth and fifth tarsometatarsal joints and talonavicular joints with pins.

**Figure 6:**
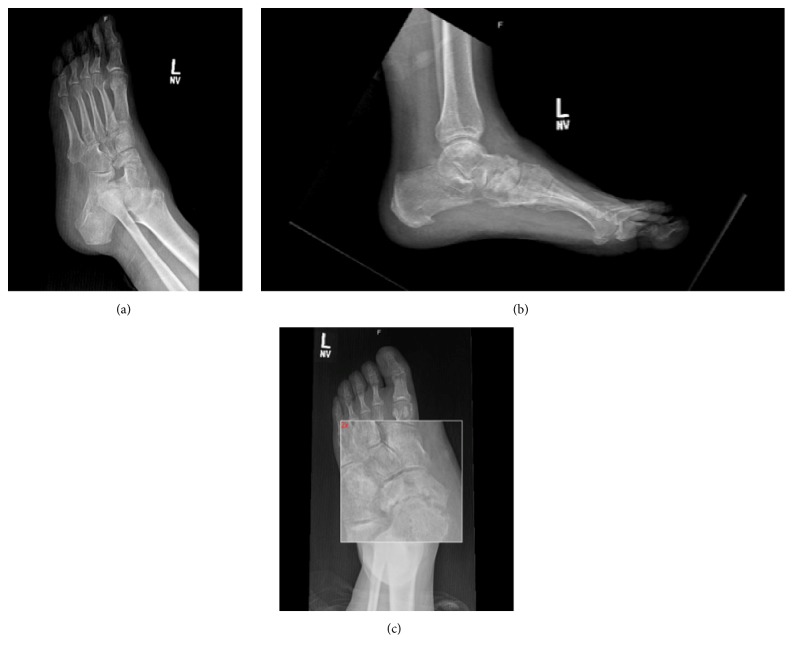
Final radiographs demonstrating development of talonavicular arthritis.

Following reduction and splinting the patient underwent operative intervention on the following day. During the procedure the fourth and fifth tarsometatarsal joints were approached via a dorsal longitudinal incision and dorsal dislocation of both joints was visualized. Reduction was obtained by manual dorsal pressure applied to the fourth and fifth metatarsal bases. A pair of 2.0-millimeter Kirschner wires was introduced percutaneously traversing both fourth and fifth metatarsal bases into the cuboid. Direct visualization as well as intraoperative fluoroscopy demonstrated an adequate reduction. Subtle instability was also demonstrated in the talonavicular joint, which was subsequently stabilized with two K-wires (Figure 4). Postoperatively the patient was placed into a well-padded short leg cast and made non-weight bearing (Figures 5(a)–5(c)). The K-wires were removed at eight weeks and he was gradually transitioned to full weight bearing in an equalizer boot. Interval radiographs demonstrated maintenance of congruent reduction of the subtalar, talonavicular, fourth, and fifth tarsometatarsal joints. At one-year follow-up, the patient had returned to his previous occupation, although he continued to experience mild to moderate pain on prolonged walking. His Foot and Ankle Disability Index (FADI) and American Orthopaedic Foot and Ankle Society (AOFAS) scores were 64 and 65 points, respectively. Radiographs obtained at one-year, however, demonstrated radiographic signs of posttraumatic arthritis at the midfoot (Figures 6(a)–6(c)).

## 3. Discussion

Subtalar dislocations are complex injuries and the final outcomes often are affected by a number of patient- and injury-related factors. A variety of bone and tendon injuries have been described in association with subtalar dislocations, many of which are identified in postreduction computed tomographic scans, emphasizing the need for obtaining advanced imaging following closed reduction. Open subtalar and total talar dislocations, in general, have less favorable prognoses with high incidences of avascular necrosis, posttraumatic arthrosis, osteomyelitis, and complex regional pain syndrome [15]. Although adequate reduction of pure ligamentous, isolated, medial and lateral, closed subtalar dislocations may potentially lead to favorable results at short- to mid-term follow-up, the presence of associated injuries often determines ultimate functional outcomes [16, 17]. We report a case of subtalar dislocation in a middle aged man with associated dislocations of fourth and fifth TMT joints and lateral cuneiform fracture. To the best of our knowledge, this combination of injuries has not been reported previously.

This injury may have potentially occurred as a result of sustained longitudinal compression and plantar flexion of the mobile lateral column of the foot following sudden eversion injury causing the lateral subtalar dislocation. It is also likely that rotation and abduction forces to the midfoot may have contributed to this unusual combination of injuries. Our patient underwent open reduction and internal fixation to stabilize the lateral column and talonavicular articulations, which resulted in a stable and plantigrade foot without radiographic evidence of talar avascular necrosis at latest follow-up.

Previous authors have stressed the importance of postreduction CT scans for critical evaluation of subtalar dislocations and defining associated injuries. Bibbo et al., in a retrospective analysis of nine cases, found that CT scans identified additional injuries in 100% of cases that were initially unrecognized on plain radiographs [14]. This was clearly emphasized in our case in which a fracture of the lateral cuneiform and base of fourth metatarsal could not be identified on plain radiographs. Additional injuries to the sustentaculum tali or lateral process of talus may cause incongruities of the subtalar joint and lead to development of painful subtalar arthritis [18]. While comminution of these articular processes may preclude fixation and necessitate excision, large isolated fragments of the posterior or lateral process may remain amenable to open reduction and internal fixation and may not affect overall outcomes negatively [11]. Therefore, early recognition of these accompanying injuries often determines further treatment plans and the long-term consequences of this relatively rare injury. Furthermore, avascular necrosis of the talus has been reported in 0 to 10% of closed subtalar dislocations, with much higher incidences up to 50% found with open dislocations. Camarda et al., in their review of thirteen patients with closed subtalar dislocation at a mean follow-up of 6 years, found that although a majority of patients had transient pain, swelling, and decreased ankle range-of-motion, this did not restrict their daily activities [17]. In addition, they reported lower AOFAS scores in patients with associated peritalar osseous injuries. Although our patient had a stable plantigrade foot with minimal deficits in hindfoot and midfoot range-of-motion and had returned to work as a laboratory technician, he had persistent problems with prolonged walking and heavy activities. At one-year follow-up, he had radiographic evidence of arthritis in the talonavicular joint without evidence of subtalar or ankle arthritis.

In conclusion, low energy closed subtalar dislocations may be associated with substantial bony and ligamentous injuries, including tarsometatarsal dislocations. Recognizing these uncommon injury patterns with postreduction CT scans is critical to determining their optimal treatment. Although early results may be reassuring, continued surveillance is necessary, as long-term outcomes may be affected by development of midfoot and hindfoot arthritis in closed subtalar dislocations with peritalar dislocations.
